# Recent Progress in DNA Biosensors: Target-Specific and Structure-Guided Signal Amplification

**DOI:** 10.3390/bios15080476

**Published:** 2025-07-23

**Authors:** Jae Eon Lee, Seung Pil Pack

**Affiliations:** Department of Biotechnology and Bioinformatics, Korea University, Sejong 30019, Republic of Korea; wodjs610@korea.ac.kr

**Keywords:** DNA assembly, signal amplification, isothermal amplification, DNA nanostructure, aptamer, DNA sensor

## Abstract

Deoxyribonucleic acid (DNA) is not only a fundamental biological molecule but also a versatile material for constructing sensitive and specific biosensing platforms. Its ability to undergo sequence-specific hybridization via Watson–Crick base pairing enables both precise target recognition and the programmable construction of nanoscale structures. The demand for ultrasensitive detection increases in fields such as disease diagnostics, therapeutics, and other areas, and the inherent characteristics of DNA have driven the development of a wide range of signal amplification strategies. Among these, polymerase chain reaction (PCR), rolling circle amplification (RCA), and loop-mediated isothermal amplification (LAMP) represent powerful target-based methods that enzymatically increase the concentration of nucleic acid targets, thereby boosting detection sensitivity. In parallel, structure-based strategies leverage the nanoscale spatial programmability of DNA to construct functional architectures with high precision. DNA can be used as a scaffold, such as DNA nanostructures, to organize sensing elements and facilitate signal transduction. It can also function as a probe, like aptamers, to recognize targets with high affinity. These versatilities enable the creation of highly sophisticated sensing platforms that integrate molecular recognition and signal amplification. Driven by DNA nano-assembly capability, both target-based and structure-based approaches are driving the advancement of highly sensitive, selective, and adaptable diagnostic technologies. This review highlights recent developments in DNA nano-assembly-driven amplification strategies.

## 1. Introduction

Deoxyribonucleic acid (DNA) is an essential molecule for all living organisms [1], including viruses [2], and it can serve as a powerful tool for biological signal detection [3]. In particular, the nanoscale self-assembly of DNA provides a universal approach for signal recognition [4]. This is enabled by DNA hybridization following the Watson–Crick base pairing rule. DNA hybridization-based sensing technology effectively detects specific target DNA [5,6]. Beyond one-dimensional hybridization, it can form higher-order structures to enhance sensing precision and efficiency [7]. As the need for ultrasensitive bioassays grows in disease diagnosis, therapeutic, environmental monitoring, and other research areas [8,9,10], advanced DNA self-assembly and hybridization techniques play a crucial role in amplifying detection signals [11,12]. This necessitates the development of novel signal amplification strategies to maximize the signal output [13].

Signal amplification is a key factor in DNA-based sensing, and various DNA assembly strategies have been employed for this purpose [14]. These strategies leverage enzyme-assisted or enzyme-free mechanisms to enhance detection sensitivity. Among them, enzyme-free methods offer advantages in simplicity, but they often face limitations in achieving ultrahigh sensitivity [15,16,17]. In particular, the detection of target DNA signals at low concentrations remains a significant challenge [18]. Notably, polymerase chain reaction (PCR) is a representative method that amplifies specific DNA sequences to overcome detection limits. PCR not only increases detection sensitivity by amplifying the desired DNA but also improves selectivity by increasing the concentration difference between the target and unwanted DNA [19]. A basic detection method for PCR products is gel electrophoresis, which allows verification with low specificity, even without sequence information [19,20]. Of the alternative methods for target amplification, rolling circle amplification (RCA) is a simple and efficient isothermal enzymatic process that utilizes a nuclease to generate long single-stranded DNA (ssDNA) or RNA [21]. PCR requires temperature cycling, whereas the RCA process is conducted at a constant temperature and requires only a small amount of circular DNA template [22]. Another isothermal method, loop-mediated isothermal amplification (LAMP), amplifies DNA with high specificity and efficiency using a set of primers to generate and use a loop structure [23]. However, simple amplification alone lacks reliability, leading to the development of strategies that further process amplified products to enhance accuracy [24,25,26].

Recently, along with these powerful amplification techniques, sensing approaches utilizing DNA-based structures have gained significant attention. Representative DNA-based structures include aptamers and DNA nanostructures. While both leverage the self-assembly properties of DNA, they differ in their purposes and functions. Aptamers, often referred to as “chemical antibodies,” are single-stranded DNA or RNA oligonucleotides that bind to specific targets with high affinity and selectivity [27,28,29]. Their intrinsic advantages, such as easy synthesis, convenient modification, and good biocompatibility, make them highly effective probes for detecting DNA, RNA, proteins, and small molecules [30]. Accordingly, aptamers exhibit strong purposefulness as probes. In contrast, DNA nanostructures can be used as programmable structural materials [31]. DNA strands contain chemically tunable groups enabling easy functionalization with molecules and nanomaterials [32]. As a result, DNA nanostructures brought rich structural and functional diversity [33]. By serving as nucleic acid-based scaffolds, they overcome the limitations of conventional DNA-based sensing, which relies on the random diffusion of free DNA [34]. Furthermore, DNA nanostructures allow easy modification of nucleic acid probes, providing diversity for DNA probe development [34,35]. DNA nanostructures and aptamers possess distinct yet complementary functionalities, making them well-suited for combined use. As scaffolds, DNA nanostructures are often utilized alongside aptamers to generate synergistic effects, leading to enhanced performance, as demonstrated in numerous studies.

By integrating structural assembly and amplification strategies, DNA-based sensing is evolving into a highly precise and versatile diagnostic platform. With its expanding applications in biosensors, medical diagnostics, and targeted therapeutics, the importance of DNA sensors continues to grow. This review will focus on how DNA nano-assembly-based signal amplification enhances the accuracy and sensitivity of DNA sensing.

## 2. Target-Based Signal Amplification

Nucleic acid biomarkers play a crucial role in disease diagnostics because DNA and RNA can be amplified from trace amounts and detected through complementary base pairing [36]. Nucleic acid amplification involves the enzymatic replication of a target nucleic acid sequence, followed by detection using techniques such as PCR, RCA, LAMP, and others [37,38]. There has been widespread adoption of target-based amplification sensing (Table 1).

### 2.1. Polymerase Chain Reaction (PCR)

Today, PCR stands as one of the most essential and extensively used tools in biosciences, diagnostics, and forensic science [51]. PCR offers programmability and precise control through the design of primers, DNA structure, cycling parameters, and the amount, length, and sequence [52]. The method relies on Taq DNA polymerase, a thermostable enzyme that allows it to remain active during repeated heating cycles, which is essential for DNA denaturation and contributes to improved primer specificity [53]. PCR involves the repetition of three key steps: denaturation, annealing, and extension. During denaturation, the double-stranded DNA template is converted into single strands by heating. In the annealing step, the reaction is cooled to allow primers to hybridize to their complementary target sequences. Finally, in the extension step, DNA polymerase binds to the primers and synthesizes new DNA strands. Each step is controlled by specific temperatures and durations, and repeated cycling of this process enables the exponential amplification of the target DNA fragment. The strategic design of primers and structures has extended the application of PCR beyond traditional amplification, enabling its integration into various sensing platforms, while other factors further enhance the accuracy and sensitivity of the sensor.

For example, a study demonstrated that a G-quadruplex-containing sequence can serve as a primer, while the target functions as a template to achieve sensing [42]. Principally, the primer sequences remain in a hairpin structure to conceal the G-quadruplex structure for interacting with the target DNA, herein referred to as hepatitis B virus (HBV) DNA. It follows elongation and leads to the opening of the hairpin structures. The exposed G-quadruplex, in the presence of K^+^, specifically interacts with an iridium (III) complex. This results in an enhanced emission response proportional to HBV DNA concentration. This method follows the fundamental principles of PCR, detecting target sequences by translating elongation-induced structural changes into measurable signals. Notably, this method achieves a remarkable sensitivity down to 1.6 fM. Another PCR-based detection approach involves precise temperature modulation of the amplification steps through plasmonic photothermal (PPT) effects. The PPT PCR system uses metallic nanoparticles to convert light into heat, enabling the precise control of thermal cycling steps without conventional thermocyclers [40,41,54]. This technique integrates PPT heating with a colorimetric detection system for fast, cost-effective, and quantitative detection of nucleic acids. In this system, these temperatures are precisely regulated by adjusting the intensity of the irradiation. Furthermore, the study uses irradiation on SYBR Green I-bound dsDNA to generate reactive oxygen species (ROS), which subsequently catalyze the oxidation of 3,3′,5,5′-tetramethylbenzidine (TMB) for color change. Consequently, when the target (λ-DNA in this study) is detected as a primer, irradiation-induced PCR proceeds. It converts the template into dsDNA and allows SYBR Green I to intercalate. The bound SYBR Green I then facilitated ROS generation, ultimately leading to a colorimetric change via TMB oxidation for target sensing. As a result, the LOD was determined to be 63.7 aM.

As discussed above, certain approaches incorporate strategic modifications into the PCR amplification process itself to enable sensing functionality. However, there are also cases where signal amplification strategies are applied to the detection of PCR products to enhance sensitivity and analytical performance. In this context, studies have demonstrated that accuracy and sensitivity in PCR-based sensing can be further improved by enhancing signal output strategies. Specifically, Surface-enhanced Raman Scattering [41] and Printed Circuit Board electrodes [39] have been utilized to amplify detection signals, enabling more precise and sensitive nucleic acid sensing. Consequently, these approaches have achieved LOD values as low as 3.12 pg/μL and 10^4^–10^5^ copies/μL, respectively.

### 2.2. Rolling Circle Amplification (RCA)

RCA is a simple and highly efficient isothermal enzymatic strategy that generates ultralong single-stranded DNA from minimal circular template, benefiting from mild conditions and stability in complex biological environments [22]. RCA is based on its ability to continuously synthesize DNA under isothermal conditions, in contrast to the thermal cycling required in PCR. This difference arises from the strand displacement activity of Phi29 DNA polymerase used in RCA, which can autonomously unwind the DNA template without the need for high temperatures [55]. In a typical RCA reaction, a primer is annealed to a circular DNA template, and the reaction is maintained at a constant temperature. As the polymerase moves around the circular template, it synthesizes a complementary strand and displaces the previously synthesized DNA, leading to the continuous generation of long single-stranded DNA composed of tandem repeats of the template sequence. Due to its high amplification efficiency and specificity, RCA has been widely recognized as a powerful tool in biosensing applications [56].

One study devised a target-specific rolling circle amplification (RCA) system for the detection of *Klebsiella pneumoniae* by exploiting the enzymatic activity of a species-encoded topoisomerase IV (Topo IV) [46,57]. Instead of relying on externally prepared circular DNA templates, the strategy employed hairpin-shaped DNA substrates immobilized on a surface, which were selectively cleaved and ligated by K. pneumoniae Topo IV to generate the required circular templates in situ [46]. These amplified products served as scaffolds for the hybridization of fluorescently labeled probes, resulting in a highly amplified signal. Consequently, this approach achieved LOD of 70 colony-forming units/μL. Just as the mentioned study achieved sensing through template design, other studies have focused on strategic primer modifications. Among these, one approach involved combining primers with aptamers to induce RCA [45]. The target interacts with the aptamer, triggering a structural change that initiates amplification. To generate a detectable signal, a probe conjugated with urease was designed to interact with the RCA product. The enzymatic activity of urease induced a pH change, enabling litmus-based sensing. As a result, this method successfully detected thrombin, platelet-derived growth factor (PDGF), and SARS-CoV-2, achieving LOD values of 50 pM for thrombin, 5 pM for PDGF, and 3.2 × 10^3^ copies/mL for SARS-CoV-2. By integrating molecular recognition, amplification, and signal transduction into a single platform, this approach simplifies the sensing workflow while maintaining versatility across diverse targets. Moreover, the simplicity of pH-based colorimetric detection suggests its scalability and adaptability for point-of-care diagnostics.

As well as designing strategies based on templates and primers, some studies focus on RCA products to amplify the signal. Typically, this involves fragmenting the RCA-generated products to facilitate signal output. One such approach accumulated these fragments and connected them with an electrochemical DNA (E-DNA) sensor [43]. E-DNA sensors operate by immobilizing a single-stranded probe on a recognition layer, where target DNA binds through base-pairing interactions [58]. This recognition event is then transduced into an optical, mechanical, or electrochemical signal [58]. In this study, the RCA products with long tandem repeats were cleaved into many small monomers by introducing a restriction endonuclease. These monomers then served as secondary targets, triggering the E-DNA sensor and generating an amplified redox current. This is an example that leverages the inherent property of RCA to generate long single-stranded DNA composed of tandem repeats of a uniform sequence. Similarly, another approach harnessed this repetitive sequence characteristic of RCA GelRed, methylene blue, and gold nanoparticles as signal reporters [47]. This strategy enhances signal intensity by facilitating the accumulation of multiple reporter molecules along the tandem repeats generated by RCA. To recognize the repeated sequences, the researchers took intrinsic features of CRISPR/Cas systems, which have been shown to be exceptional tools for specific nucleic acid detection, such as those associated with diseases [59,60]. In this approach, an RCA product-based hydrogel was designed to encapsulate signal molecules. CRISPR/Cas12a was programmed to recognize both the target DNA and the RCA product, forming a CRISPR-responsive RCA hydrogel system. Upon the introduction of the target DNA, the hydrogel structure disintegrated and led to the release of signal molecules. Furthermore, by using different signal molecules, the signal output could be customized, enabling fluorescence, electrochemistry, and colorimetry, thereby ensuring adaptability to various detection scenarios. Notably, this platform achieved a LOD as low as 10 copies/μL, demonstrating its high sensitivity.

### 2.3. Loop-Mediated Isothermal Amplification (LAMP)

LAMP is a fast, specific, and cost-effective isothermal method that enables self-sustained sequence replication and strand displacement of nucleic acid [61]. Principally, this method uses specially designed primers to form self-priming stem-loop DNA structures, enabling continuous strand displacement and amplification [62]. Once the initial structures are formed, primers anneal to the loop regions and initiate DNA synthesis. As the polymerase extends the strand, strand displacement pushes aside the newly synthesized DNA. This results in the continuous generation of longer amplification products with multiple stem-loop motifs. These products adopt dumbbell-like conformations that function as templates for further amplification, ultimately yielding DNA strands composed of repeated target sequences and characteristic secondary structures [62]. Coupled with easy detection techniques of amplicons, LAMP provides a simple-to-operate and easy-to-read molecular diagnostic tool, enabling the development of various LAMP-based diagnostic kits and assays targeting diverse pathogens [63].

**Figure 1:**
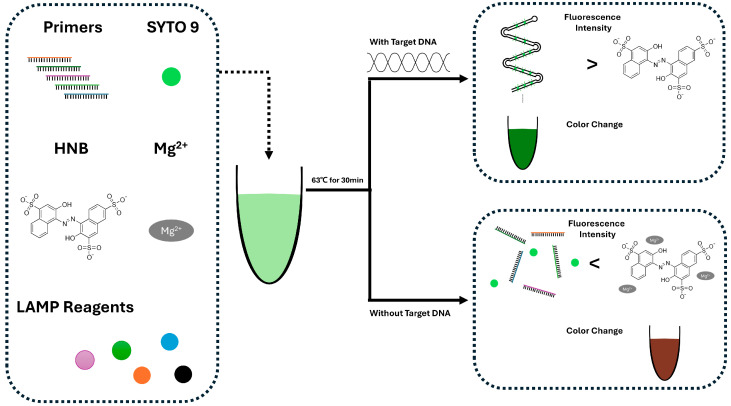
**Scheme of the dfLAMP (dual-color fluorescence LAMP) assay.** Before amplification, sufficient Mg^2+^ allows HNB to emit weak red fluorescence. At room temperature, LAMP primers may form a small amount of non-specific dsDNA, which binds SYTO 9. As a result, both positive and negative samples show slight green fluorescence. In positive samples after amplification, large amounts of magnesium pyrophosphate form, reducing Mg^2+^ concentration and leading to a decrease in red fluorescence. Simultaneously, abundant dsDNA amplicons with SYTO 9 produce strong green fluorescence. In negative samples, the non-specific dsDNA could become denatured at the reaction temperature, leading to reduced SYTO 9 binding and green fluorescence. Red fluorescence from HNB recovers due to the retained Mg^2+^, resulting in a red signal. Redrawn from [49].

From this perspective, there is a study that combines LAMP with easy-to-read fluorescent molecules [49] (Figure 1). In this study, SYTO 9 and hydroxyl naphthol blue (HNB) were used together to detect fluorescence signals during LAMP-based DNA amplification. The fluorescence intensity difference between amplified and non-amplified samples allowed clear visualization. In the presence of target DNA, SYTO 9 binds to the amplified DNA products generated using LAMP, resulting in increased fluorescence. Simultaneously, Mg^2+^ ions are sequestered by the elongated DNA strands, minimizing their interaction with HNB and maintaining baseline absorbance. In contrast, in the absence of the target, DNA amplification does not occur, leaving free Mg^2+^ ions available to bind with HNB. Thereby, it produces a distinct colorimetric (negative) signal. This enabled the development of a dual-color fluorescence LAMP (dfLAMP) assay, which can detect as little as 1 fg of Atlantic salmon DNA. In contrast to methods that detect LAMP-derived amplicons, an alternative strategy directly utilizes biochemical changes within the amplification process. For example, a study performed detection based on the intrinsic pH shift generated during the LAMP reaction itself [48]. In this study, the authors observed a pH decrease in a low-buffered LAMP reaction [64] and utilized this pH change to determine whether the LAMP reaction had occurred. This pH shift was detected using ion-sensitive field-effect transistors (ISFETs) by measuring voltage variations. A LOD of 10^3^ copies/mL was achieved for *Mycoplasma pneumoniae* (MP). However, this method merely detects the pH change that occurs during the LAMP reaction. It may be difficult to distinguish whether the observed pH shift is caused by target-specific amplification or by non-specific background activity.

While successful sensing like dfLAMP or pH-based LAMP-ISFETs assays are achievable, achieving both high sensitivity and specificity simultaneously in LAMP is challenging. It may result in false positives since improving specificity can lead to a reduction in sensitivity [24]. To address this, studies have been reported that simultaneously enhance sensitivity by using LAMP-based amplification and accuracy by serving the amplification product as a trigger. One approach involved designing the LAMP process such that amplification led to the release of a DNA trigger [24]. This trigger then served as the initiator for downstream signal generation. In this study, the amplification process was designed so that the trigger is naturally released during the LAMP process. This released trigger subsequently initiates signal generation, enabling specific signal filtration. As a result, it was possible to detect target DNA as low as 5 copies per reaction and RNA as low as 10 copies per reaction with high specificity. Another study serves as an amplification of products as triggers for the CRISPR/Cas system [50]. In this study, the CRISPR/Cas system acts as a programmable molecular scissor. Specifically, upon binding of the CRISPR-Cas12a complex with the amplified target DNA strand, the Cas12a protein undergoes conformational changes that activate its nonspecific endonuclease activity. It makes cleavage of nearby single-stranded DNA (collateral cleavage) [36,50]. They used this phenomenon and created a single-stranded DNA probe labeled with both a quencher and a fluorescent molecule. Using this system, the SARS-CoV-2 RNA of envelope protein (E) and nucleocapsid protein (N) were detected, achieving a LOD of 50 copies/μL, and were successfully applied in clinical samples. To enhance the amplification efficiency, a recent study has reported the combination of LAMP and RCA to improve amplification performance [65]. Traditional RCA is a linear signal amplification mechanism, which often results in limited amplification efficiency. For better efficiency, LAMP has been integrated with RCA for microRNA detection. In this study, the product generated by RCA was used as a template for LAMP. Due to the repetitive nature of the RCA product, multiple LAMP reactions could occur simultaneously on identical sequence motifs, resulting in a high amplification efficiency. This combination significantly enhances both amplification efficiency and detection sensitivity. As a result, even microRNA targets as low as 10 aM can be clearly and accurately detected.

## 3. Structure-Based Signal Amplification

Methods that amplify signals (such as PCR, RCA, LAMP, etc.) principally rely on target sequences to generate signals, making them advantageous for detecting specific sequences. However, their sensitivity may decrease whenever sequence mutations occur. In contrast, structure-based sensing conjugates specific structural assemblies with molecular interactions to recognize targets. It allows the detection of various biomolecules, including simple sequences [66], proteins [67], virus [68,69], small molecules [70,71,72,73], and even metal ions [74,75], making it less susceptible to sequence variations and enhancing overall detection robustness. Moreover, by leveraging the characteristics of the formed structures, sensing platforms can be tolerant to various environmental changes [76]. Accordingly, sensing based on structural properties has been widely used (Table 2). In this chapter, we discuss how structure-based signal amplification enhances sensitivity and selectivity.

### 3.1. DNA Nanostructure

DNA nanostructures are highly programmable nanoscale materials that use predictable base pairing rules to enable the self-assembly of single strands into predesigned, precise, and complex shapes for diverse applications [99,100,101]. Sensing applications using DNA nanostructures fundamentally rely on their ability to transduce molecular interactions into detectable signals, typically through target-induced changes in size, conformation, or conductivity. Also, it allows enhancing biosensing performance from the high surface area of nanostructures and the high loading of recognition elements with spatially controlled placement, thus improving charge-sensitive conductance [102,103]. Accordingly, several studies have reported biosensing strategies triggered by target sequences.

**Figure 2:**
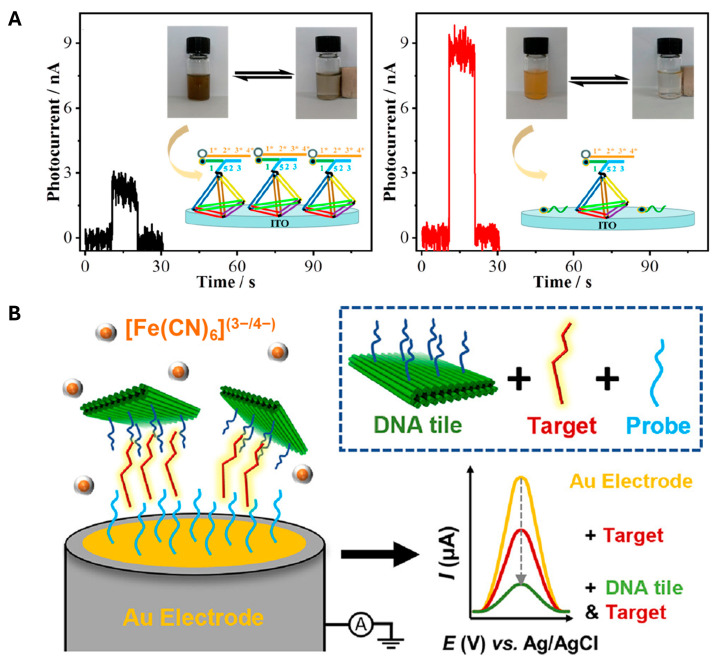
**Representative examples of DNA nanostructure-based biosensors** (**A**) Photoelectrochemical (PEC) biosensor utilizing a DNA tetrahedron structure for miRNA-196a detection, demonstrating signal change in the absence and presence of 5 fmol L^−1^ target. Reprinted with permission from [81]. Copyright © 2021 American Chemical Society. (**B**) Signal amplification strategy based on DNA origami tiles. DNA origami (green) bearing capture strands (deep blue) hybridizes with target strands (red), which are further anchored onto ssDNA-probe (light blue)-functionalized polycrystalline gold electrodes (PGE). Thereby, it modulates redox species distribution. Reprinted from [77] licensed under CC BY 4.0. Copyright © 2023 Williamson et al. Published by American Chemical Society.

As an example, Li et al. detected miRNA-196a using an immobilized DNA nanostructure as a conductor [81]. In this study, a DNA tetrahedron was immobilized on the sensing platform. The structural response was triggered by the target sequence, leading to the removal of the sequence conjugated with the magnetic particle. This structural transformation caused a noticeable visual change, as illustrated in the right panel of Figure 2A. Based on this change and the photoelectrochemical sensing mechanism, the presence of the target sequence could be reliably determined. As a result, the platform’s conductivity slightly changed, achieving a 3.1 amol/L LOD value. Similarly, the immobilizing method enabled a change in conductivity via enzyme-mediated cleavage [78,82]. In other cases, DNA nanostructures are anchored by target sequences [77] (Figure 2B). In this approach, probe sequences were immobilized to hybridize with specific target sequences. Researchers designed DNA origami structures that precisely matched the target, enabling accurate recognition on the sensing platform. This design enhanced the platform’s specificity by preventing nonspecific interactions and achieved a limit of detection of 8.86 pM in the presence of the target. These examples collectively demonstrate how the fundamental sensing principles of DNA nanostructures, which undergo structural and size changes in response to targets, can be effectively utilized for reliable and sensitive detection.

**Figure 3:**
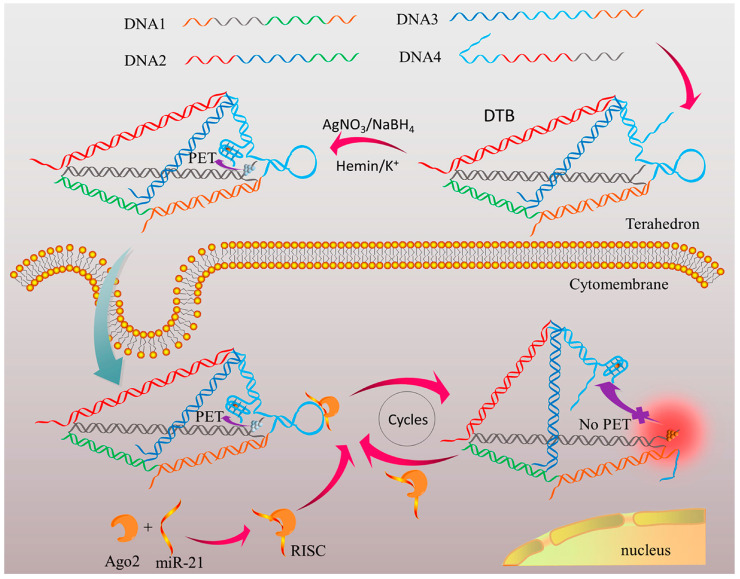
**Principle of Ago2 assay in a single cell using the DTB (DNA tetrahedron-based biosensor).** A functional edge of the DNA tetrahedron carries a G-quadruplex (G4) motif and a silver nanocluster (AgNC) fluorescence reporter. In the absence of a target, a hairpin structure brings these motifs into proximity, quenching the signal via PET. Upon recognition and cleavage by the Ago2/miR-21 complex, the tetrahedron undergoes conformational change. Consequently, the motifs separate and restore fluorescence, enabling specific detection of Ago2 activity. Reprinted with permission from [83]. Copyright © 2019 American Chemical Society.

Besides the changes in conductivity induced by target-triggered size changes, other studies have explored signal amplification through enzyme-mediated sensing [79,80] or fluorescence-based sensing [83,104]. Unlike conventional DNA nanostructure-based sensors that primarily detect nucleic acids, there are also cases where DNA nanostructures sense proteins with some limitations. For example, one of the studies designed a DNA tetrahedron-based biosensor (DTB) for imaging and detection of argonaute2 (Ago2) via photoinduced electron-transducer (PET) [83] (Figure 3). In the DTB, one edge strand of the tetrahedron contains a hairpin-structured sequence that interacts specifically with miR-21 bound to Ago2. Another DNA strand is modified at both ends with two PET pairs: a DNA/silver nanocluster (AgNC) and a G-quadruplex/hemin complex, respectively. In the absence of the Ago2/miR-21 complex, these PET pairs are held in close proximity, causing fluorescence quenching. However, when the complex is present, the hairpin is cleaved, triggering a conformational change that spatially separates the PET pairs. This separation restores AgNC fluorescence, enabling both quantitative detection and even cellular imaging. This approach demonstrates a way for DNA nanostructures to overcome certain sensing limitations by detecting proteins without additional probes. Nonetheless, it faces challenges in detecting a broader range of targets, limiting its applicability.

Thus, while DNA-based platforms can be extended to some target proteins, their applicability remains limited to DNA, RNA, and a subset of proteins that naturally interact with nucleic acid sequences. Even when the targeting scope is expanded by conjugating recognition elements such as antibodies [105], this often requires additional chemical modifications [106,107,108], adding complexity to the system. Therefore, there is a growing need for recognition elements that are inherently compatible with DNA nanostructures and can be incorporated without complex chemistry. Among such elements, aptamers represent one of the most suitable candidates, offering sequence-defined recognition and structural versatility within DNA frameworks.

### 3.2. Aptamer with DNA Nanostructure

**Figure 4:**
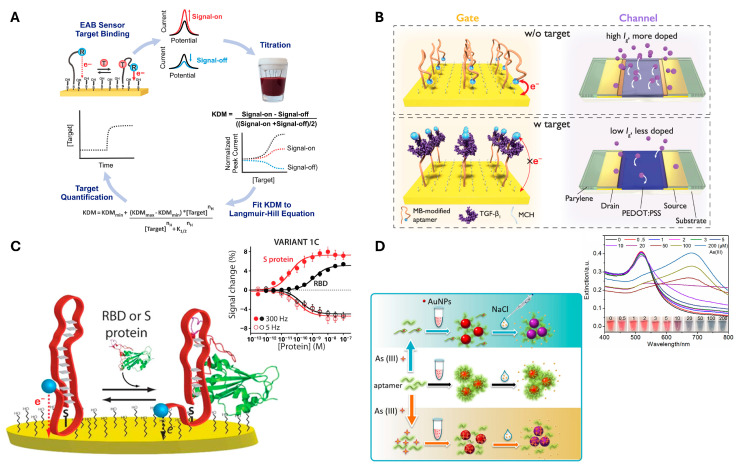
**Representative examples of Aptamer-based biosensors** (**A**) Electrochemical aptamer-based (EAB) sensors detect target binding by measuring changes in electron transfer between a redox-reporter-modified aptamer and a gold electrode using square wave voltammetry. Adapted from [73]; licensed under CC BY 4.0. Copyright © 2022 Downs et al. Published by Springer Nature. (**B**) The referenced organic electrochemical transistors (OECT)-based EAB sensor detects TGF-β_1_ by measuring changes in gate current caused by the conformational shift of the aptamer and movement of the methylene blue reporter. Adapted from [67]; licensed under CC BY 4.0. Copyright © 2023 Ji et al. Published by Springer Nature. (**C**) The EAB sensor detects SARS-CoV-2 RBD and S protein with Langmuir binding curves, showing signal-on at higher frequencies and signal-off at lower frequencies for clinically relevant detection. Adapted from [69]; licensed under CC BY 4.0. Copyright © 2021 Idili et al. Published by American Chemical Society. (**D**) As (III) sensor based on DNA adsorption on AuNPs for enhanced colloidal stability. The aptamer−As (III) interaction induces AuNP aggregation, with mechanisms varying depending on As (III) concentration and salt presence. Adapted with permission from [86]. Copyright © 2019 American Chemical Society.

Aptamers are constructed based on base-pairing rules to form a nanoscale self-assembled three-dimensional structure, forming double-stranded regions and single-stranded loops within the molecule. These loops form precise spatial motifs that facilitate high-affinity and specific binding to a concatemeric ligand [109]. Importantly, aptamers offer exceptional flexibility and convenience in structure design, paving the way for innovative biosensors that demonstrate high sensitivity and selectivity [110]. Aptamers typically utilize target-induced structural changes to perform sensing. These changes can alter the distance to an electrode, leading to changes in conductivity. Such studies have enabled the detection of various targets, including TGF-β [67], the SARS-CoV-2 spike protein [69,111], as well as small molecules such as ATP [112], vancomycin [73], ampicillin [70], tryptophan [71], and doxorubicin [72] (Figure 4). Remarkably, even metal ions [75] like Hg^2+^ and Pd^2+^ have also been successfully detected using this approach.

Furthermore, compared to using aptamers alone, the integration of DNA nanostructures into aptamer-based sensors offers several advantages, such as improved spatial organization [113,114], enhanced stability [115,116], higher sensitivity [117,118], and minimized false positives [119]. In addition, since both aptamers and DNA nanostructures are composed of nucleic acids, they can be easily modified simply by hybridization. For example, Li et al. [87] utilized a DNA tetrahedron to precisely control the spatial distance between aptamers and the electrode, which enhanced signal efficiency. By introducing a one-base pair mismatch, they were also able to effectively reduce background signals. In another study, Suo et al. [119] employed quencher sequences closely positioned around the aptamer using dual dual-cross DNA nanostructure. Thereby, this minimized background in the absence of the target (off-state). In these examples, not only were the advantages of integration effectively applied, but the incorporation of aptamers also contributed to expanding the range of detectable targets.

Building upon these concepts, other studies have successfully realized sensing by harnessing specific molecular phenomena, such as fluorescence quenching. Among the diverse strategies enabled by integrated systems, dual fluorescence-based approaches have attracted considerable interest due to their capacity for ratiometric sensing and multiplexed target detection [120]. One approach employs a dendrimer-like DNA nanostructure coupled with catalytic hairpin self-assembly to construct an amplifiable ratiometric fluorescent aptasensor for aflatoxin B1 (AFB1) [88]. The branched scaffold not only enhances fluorescence resonance energy transfer (FRET) efficiency but also enables signal amplification through target-triggered cyclic assembly, offering improved sensitivity (5 pg/mL) and a wider linear range. Another design features a tuning fork-shaped DNA (TF-DNA) architecture that stably anchors two distinct fluorescently labeled aptamers for AFB1 and ochratoxin A (OTA) [89]. Upon target binding, the aptamers dissociate from the TF-DNA duplex, releasing fluorescence signals that can be simultaneously quantified. This structural design ensures mechanical stability and supports dual-target detection (OTA: 0.015 ng/mL, AFB1: 0.045 ng/mL) with high specificity. These results were largely attributed to the precise spatial control provided by DNA nanostructures and the target-triggered conformational changes induced by aptamers. This combination can significantly contribute to the implementation of distance-sensitive mechanisms, such as FRET, where precise spatial control is essential.

**Figure 5:**
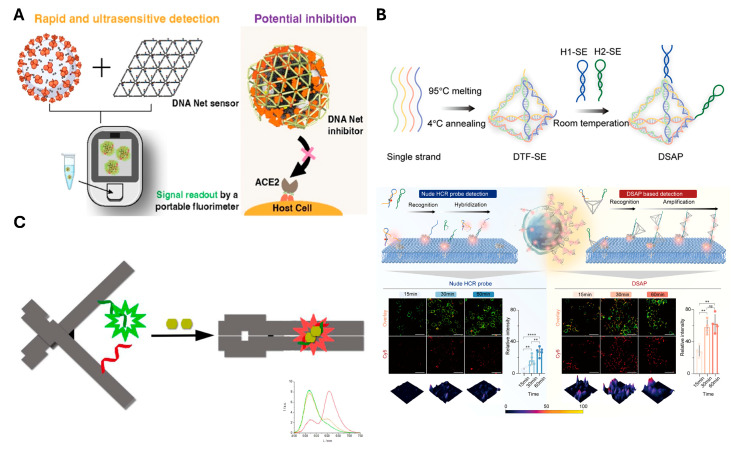
**Representative examples of sensor combining aptamer with DNA nanostructure** (**A**) Rationally designed DNA Net incorporates spike RBD-targeting aptamer for promoting high-affinity multivalent binding. The mechanism uses FAM-labeled aptamers quenched, which are released upon viral binding to generate a fluorescence signal. The sensing platform complex also blocks spike interactions to enable potential virus neutralization. Reprinted with permission from [97]. Copyright © 2023 American Chemical Society. (**B**) Principle and performance of high-throughput immune monitoring with DNA framework signal amplification platform (DSAP). The structure is assembled from four DNA strands and connected to H1-SE and H2-SE, which include aptamers, via sticky ends. CLSM images compare membrane protein detection efficiency between DSAP and conventional (nude) HCR probes. A quantitative comparison of signal intensities is shown in the accompanying histogram. The asterisks denote statistical significance, with more asterisks indicating higher levels of significance. Adapted from [98] licensed under CC BY 4.0. Copyright © 2022 Chen et al. Published by Springer Nature. (**C**) DNA origami-based sensor incorporates a split aptamer labeled with a FRET dye pair, enabling ATP-induced conformational change upon target binding. Adapted with permission from [93]. Copyright © 2017 American Chemical Society.

Extending this concept of delicately engineered sensors, larger and more complex DNA nanostructures with aptamers have been developed to enhance target recognition and diagnostic performance. A net-shaped DNA nanostructure (called “DNA Net” herein) is designed to geometrically align multiple spike-targeting aptamers with the trimeric spike protein arrangement on SARS-CoV-2 virions [97] (Figure 5A). The spatial arrangement of target proteins on the virus was mimicked by the Net, with aptamers positioned at matching intervals. This design enabled the DNA Net to wrap around the virus, promoting multivalent binding. As a result, the system achieved PCR-comparable sensitivity, rapid detection within 10 min, and approximately 1000-fold enhanced viral inhibition compared to monomeric aptamers. In parallel, the DNA framework signal amplification platform (DSAP) employs a modular assembly of the DNA tetrahedron that presents aptamers with high spatial density and controlled orientation for immune cell surface marker recognition [98] (Figure 5B). The DSAP was designed with two aptamers arranged in a loop configuration, enabling interaction with target proteins on the cell membrane. Upon target binding, the loops undergo structural opening, allowing the two aptamers to hybridize with one another. Importantly, unpaired regions were intentionally introduced within the hybridized structure to serve as binding sites for additional DSAP units. This design facilitates signal cascading through sequential DSAP hybridization, ultimately leading to signal amplification. As shown in Figure 5B, this system enabled significantly greater signal amplification compared to simple single-stranded probes. This structural and functional integration allows accurate immune monitoring in under 30 min with a LOD (1 cell/μL) and demonstrated clinical performance with AUC > 0.97 in HIV patient staging. Additionally, changes in electrical conductivity have also been widely exploited in sensing platforms. Target binding often induces conformational changes in the aptamer or its surrounding nanostructure. Thereby, it modulates the distance to electrodes [121] or alters charge transport properties [122]. While changes in size resulting from target binding can directly affect conductivity [90,91,92], in some cases, target recognition leads to structural transformations of the DNA nanostructure, which also modulate electrical properties. A notable example of such structural switching involves fluorescence modulation induced by a split aptamer integrated into a DNA origami [93] (Figure 5C). Upon binding to its target, herein targeted ATP, the split aptamer reassembles. That recognition induces the DNA origami to transition from open to closed form. This conformational change brings two cyanine–styryl dyes into proximity, which are positioned opposite the DNA origami. As a result, wavelength shifts occur from green to red through energy transfer, achieving high accuracy by minimizing background signals. In this way, the expansion of structural complexity or the implementation of dynamic conformational switching within DNA nanostructures allows for more distinguishable signal generation, supporting highly sensitive and specific biosensing.

Another class of examples involves the use of aptamers as building blocks of DNA nanostructures, where target recognition triggers conformational changes. In one study, aptamers were incorporated into the framework of a DNA tetrahedron, leading to a target-induced structural transformation [94]. PicoGreen, which selectively binds to double-stranded DNA (dsDNA), was employed to monitor the change. Upon target recognition, parts of the tetrahedral structure converted into single-stranded regions. This single form caused a reduction in the PicoGreen binding and thus enabling detection. This approach achieved LOD as low as 0.135 nM for OTA. Similarly, another study employed aptamers as components of DNA nanostructures, where aptamer folding facilitated the reassembly of split G-quadruplex sequences [95]. In this design, a DNA tetrahedron was functionalized on one of its edges with both an aptamer and two split G-quadruplex sequences. When the platform receives target recognition, the aptamer undergoes folding and effectively shortens one edge of the tetrahedron. This conformational change brought the split G-quadruplex sequences into close proximity, enabling the formation of a complete G-quadruplex structure capable of binding hemin. The resulting G-quadruplex–hemin complex exhibited a characteristic redox signal, which was detected using differential pulse voltammetry. As a result, it achieves a LOD as low as 50 pM for ATP. These cases demonstrate that aptamers are not limited to serving solely as target recognition elements. Instead, they are incorporated as structural components of the DNA nanostructure, enabling more pronounced and dynamic conformational changes upon target binding.

Comprehensively, aptasensors have demonstrated significant improvements in sensitivity and selectivity through integration with DNA nanostructures. These advances have expanded the potential of aptasensors across a variety of analytical platforms. Notably, there are also instances where the synergistic integration of aptamers and DNA nanostructures is coupled with pre-amplification strategies. Such approaches include the use of RCA, which can be applied either to generate template strands for assembling DNA nanostructures [123,124] or to amplify the output signal [125] of aptamer-based sensors. These multifaceted approaches maximize sensor performance by combining the structural precision of nanostructures, the target specificity of aptamers, and high amplification efficiency. Looking ahead, such hybrid technologies are expected to play a pivotal role in the development of next-generation diagnostics and biosensing systems.

## 4. Concluding Remarks and Outlook

In this review, we discussed how DNA nano-assemblies amplify signals in DNA-based biosensors and enhance both the sensitivity and accuracy of sensing platforms. Notably, the inherent properties of DNA and their versatile applications have enabled the expansion of target detection beyond nucleic acids to cells, proteins, small molecules, and metal ions.

Sensing strategies can be broadly categorized into relying on target-mediated and structure-mediated signal amplification. Target-based sensing strategies are typically limited to nucleic acid targets. They can also be susceptible to even minor sequence mutations due to their reliance on specific hybridization. Nevertheless, they offer notable advantages. These strategies are highly effective for detecting low-abundance analytes. The precise design of primers for target assembly enables enhanced sensitivity and improved limits of detection (LOD), making them powerful tools in molecular diagnostics.

Structure-based approaches extend the range of detectable targets and allow for easy integration through modular modification. These structural systems offer advantages such as spatial organization, stability, and sensitivity. That comes up presenting opportunities to overcome the limitations of conventional sensing strategies and paving the way for high-performance sensors. However, a key limitation of structure-based sensing is its relatively low signal amplification capacity compared to target-based strategies. Because structural systems often rely on conformational changes rather than exponential amplification, their sensitivity can be restricted. To overcome this, recent efforts have focused on hybrid strategies that combine target amplification with structural assembly to enhance both signal strength and specificity.

**Figure 6:**
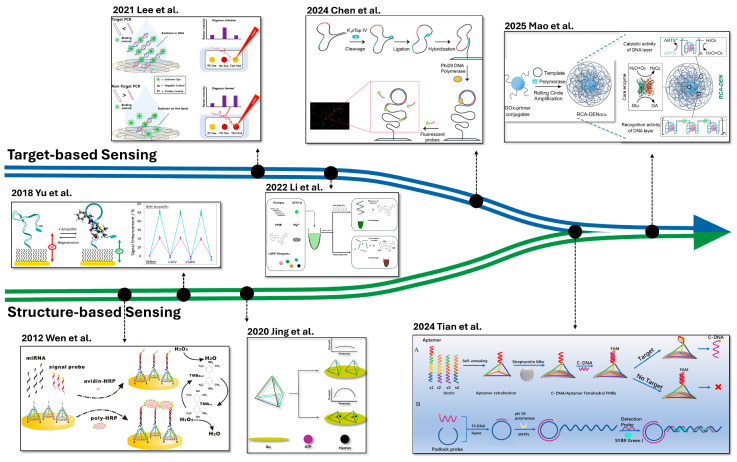
**Timeline of Development in Target- and Structure-based Sensing and Their Synergistic Integration.** Figure reprinted and redrawn from published studies (see references below) to illustrate the chronological development and integration of sensing strategies. The graph of DNA Tetrahedron Sensor using HRP (Wen et al.) is reprinted with permission from [79]. Copyright © 2012 Springer Nature. The graph of the ampicillin target aptamer sensor (Yu et al.) is reprinted with permission from [70]. Copyright © 2018 Elsevier. The graph of the switchable tetrahedral DNA sensor (Jing et al.) is redrawn from [95]. The graph of the PCR-based paper sensor (Lee et al.) is printed with permission from [41]. Copyright © 2021 Elsevier. The graph of the dual-color fluorescence LAMP assay (Li et al.) is redrawn from [49]. The graph of the RCA-based DNA sensor (Chen et al.) is reprinted from [46] with permission. Copyright © 2024 Elsevier. The graph of the Structure-initiated RCA sensor (Tian et al.) is reprinted from [125] with permission. Copyright © 2024 American Chemical Society. The upper part (A) illustrates the biosensor composed of aptamer and DNA nanostructure that produces c-DNA output. The lower part (B) depicts the amplification of the c-DNA output by RCA. The graph of the RCA-driven DNA nanostructure sensor (Mao et al.) is reprinted from [123] with permission. Copyright © 2025 Elsevier.

More recently, the boundary between target- and structure-based sensors has become increasingly blurred, with emerging hybrid strategies that synergistically combine the strengths of both (Figure 6). For example, nucleic acid targets can be amplified during the pre-amplification to serve as building blocks for nanostructure formation. These target-derived structures facilitate signal generation during the post-amplification stage through specific target recognition. Such integration not only enhances detection performance but also offers a versatile platform adaptable to a wide range of analytes.

These integrated approaches represent a promising direction for the future of DNA-based biosensors, offering new possibilities for the sensitive and specific detection of a wide array of biological and chemical targets.

## Figures and Tables

**Table 1:** The list of amplification-based sensing.

Method	Target	Transducer	Biosensing Method	Dynamic Range	Limit of Detection	Ref.
Polymerase Chain Reaction (PCR)	Nucleocapsid gene from SARS-CoV-2 isolate Wuhan-Hu-1 [DNA]	Printed Circuit Board (PCB)	Electrochemical	10 pg/μL to 200 pg/μL	10^2^~2 × 10^3^ copies/μL	[39]
	Lambda DNA	Plasmonic Photothermal Colorimetric PCR (PPT-cPCR)	Colorimetric	0.5 ng/μL to 3 fg/μL	63.7 aM	[40]
	Mycoplasma pneumoniae [DNA]	Surface-enhanced Raman Scattering (SERS)	Optical	3.12 pg/μL to 50 pg/μL	3.12 pg/μL	[41]
	Hepatitis B virus [DNA]	G-quadruplex selective iridium (III) complex	Fluorescent	3 fM to 800 pM	1.6 fM	[42]
Rolling Circle Amplification (RCA)	miR-7a [microRNA] from human serum	Electrochemical DNA (E-DNA) sensor	Electrochemical	1 fM to 100 fM	0.59 fM	[43]
	*Staphylococcus aureus* nuc gene [DNA]	Electrochemiluminescent (ECL) sensor	Electrochemical	10 aM to 1 pM	3.8 M	[44]
	Thrombin, platelet-derived growth factor (PDGF), severe acute respiratory syndrome coronavirus 2 (SARS-CoV-2) [Protein]	Aptameric sensor	Colorimetric	-	50 pM for thrombin, 5 pM for PDGF, 3.2 × 10^3^ copies/mL for SARS-CoV-2	[45]
	*Klebsiella pneumoniae* topoisomerase IV [Protein]	RCA-based DNA sensor	Fluorescent	-	70 colony-forming units (CFU)/µL	[46]
	*mecA* gene of methicillin-resistant *Staphylococcus aureus* (MRSA) [DNA]	RCA-based DNA hydrogel biosensor	Electrochemical, Colorimetric, Fluorescent	-	10 copies/μL	[47]
Loop-Mediated Isothermal Amplification (LAMP)	*Mycoplasma pneumoniae* (MP)	Ion-sensitive field-effect transistor	Electrochemical	10^3^ to 10^7^ copies/mL	10^3^ copies/mL	[48]
	African swine fever virus (ASFV) p72 gene [DNA], SARS-CoV-2 N gene fragment [RNA]	Self-replication catalyzed hairpin assembly (SRCHA)	Colorimetric	-	5 copies per reaction for DNA, 10 copies per reaction for RNA	[24]
	Atlantic salmon [DNA]	Dual-color fluorescence LAMP (dfLAMP)	Fluorescent	0.1 fg to 100 ng	1 fg of DNA	[49]
	SARS-CoV-2 RNA of envelope protein (E) and nucleocapsid protein (N)	Paper-based LAMP-CRISPR Integrated Diagnostics (PLACID)	Fluorescent	10^2^ to 10^6^ copies/μL	50 copies/μL	[50]
Polymerization/Nickase cycle	miR-200c [microRNA-200c] from prostate cancer	Catalytic Hairpin Assembly (CHA)	Fluorescent	10 fM to 10 nM	5.5 fM	[25]
Polymerase Strand Recycling (PSR)	miRNA, Tetracycline, Zinc	Cell-free biosensor	Fluorescent	-	5 nM for miRNA, 25 nM for Tetracycline, 100 nM for Zinc	[26]

**Table 2:** The list of structure-based sensing.

Template Structure	Target	Transducer	Biosensing Method	Dynamic Range	Limit of Detection	Ref.
DNA Nanostructure	*bla*OXA-1 *β-*lactamase gene sequence (DNA)	Electrochemical DNA (e-DNA) biosensor	Electrochemical	10 pM to 1 nM	8.86 pM	[77]
	miR-141	e-DNA biosensor	Electrochemical	-	31 aM	[78]
	miR-21	Electrochemical miRNAs sensor (EMRS)	Electrochemical	10 fM to 10 nM	10 fM	[79]
	Model DNA	e-DNA sensor	Electrochemical	1 pM to 1 nM	1 pM	[80]
	miRNA-196a	Photoelectrochemical (PEC) biosensor	Photoelectrochemical	10 amol/L to 10 pmol/L	3.1 amol/L (3 S/N)	[81]
	miRNA-182-5p	Paper-based PEC sensor	Photoelectrochemical	0.1 fM to 100 pM	0.09 fM	[82]
	Argonaute2 (Ago2)/miR-21, RNase H of human immunodeficiency virus type-1 (HIV-1)	DNA tetrahedron-based biosensor (DTB)	Fluorescence	-	4.54 nM for Ago2, 3.41 U/mL for RNase H	[83]
Aptamer	Ampicillin	Electrochemical aptamer-based (E-AB) sensor	Electrochemical	-	1 µM	[70]
	L-tryptophan	E-AB sensor	Electrochemical	0.7 µM to 40 µM	30 µM	[71]
	Doxorubicin	Boron-doped diamond (BDD) based E-AB sensor	Electrochemical	49 nM to 2.3 µM	49 nM	[72]
	SARS-CoV-2 spike (S) protein	E-AB sensor	Electrochemical	760 pg/mL to 76 ng/mL	760 pg/mL	[69]
	Transforming growth factor beta 1 (TGF-β1)	Referenced- organic electrochemical transistors (ref-OECTs)-based E-AB sensor	Electrochemical	1 ng/mL to 1 µg/mL	1 ng/mL	[67]
	Dopamine, Melamine	Gold nanoparticles (AuNPs) based label-free sensor	Colorimetric	-	4 μM dopamine for dopamine, 1 μM for melamine	[84]
	*Escherichia coli* (*E. coli*)	Graphene-coated AuNPs sensor	Colorimetric	10 to 10^7^ cells/mL	10 cells/mL	[85]
	Arsenic (As)	AuNP	Colorimetric	-	0.5 µM	[86]
	SARS-CoV-2 nucleocapsid (N) protein	Dual structure-switching aptamer-mediated signal amplification	Fluorescence	10 fg to 1 ng	0.59 pg/mL, 12.5 fM	[68]
Aptamer with DNA Nanostructure	ATP	DNA nanostructure-based electrochemical (E-nanoDNA) sensor	Electrochemical	10 nM to 1µM	5 nM	[87]
	Aflatoxin B1 (AFB1)	Ratiometric fluorescent aptasensor	Fluorescence	0.1 ng/mL to 50 ng/mL	5 pg/mL	[88]
	Ochratoxin A (OTA), AFB1	Fork-shaped DNA (TF-DNA) fluorescence aptasensor	Fluorescence	0.05 ng/mL to 100 ng/mL for OTA, 0.1 ng/mL to 100 ng/mL for AFB1	0.015 ng/mL for OTA, 0.045 ng/mL for AFB1	[89]
	Exosomes from hepatocarcinoma cells (HepG2)	Nanotetrahedron (NTH)-assisted aptasensor	Electrochemical	10^5^ to 10^12^ exosomes/mL	3.96 × 10^5^ exosomes/mL	[90]
	Thrombin	E-AB sensor	Electrochemical	1 pM to 1 nM	1 pM	[91]
	Vascular endothelial growth factor (VEGF)	e-DNA biosensor	Electrochemical	10 pg/mL to 100 ng/mL	5 pg/mL	[92]
	ATP	Nanomechanical DNA origami device	Fluorescence	-	-	[93]
	OTA	Fluorescent aptasensor	Fluorescence	0.3 nM to 10 nM	0.135 nM (54.5 pg/mL)	[94]
	ATP	Electrochemical aptasensor	Electrochemical	0.1 nM to 1 mM	50 pM	[95]
	Bisphenol A	Potentiometric Aptasensing	Electrochemical	0.1 nM to 100 nM	80 pM	[96]
	SARS-CoV-2	Portable fluorimeter	Fluorescence	7.32 nM to 20.26 nM	1000 viral genome copies/mL	[97]
	CD4+ T, CD8+ T lymphocytes	DNA framework signal amplification platform (DSAP)	Fluorescence	-	1/100 μL for CD4+ T cell, 4/100 μL for CD8+ T cell	[98]

## References

[1] Minchin S., Lodge J. (2019). Understanding biochemistry: Structure and function of nucleic acids. Essays Biochem..

[2] Cassedy A., Parle-McDermott A., O’Kennedy R. (2021). Virus detection: A review of the current and emerging molecular and immunological methods. Front. Mol. Biosci..

[3] Wang Q., Wang J., Huang Y., Du Y., Zhang Y., Cui Y., Kong D.-m. (2022). Development of the DNA-based biosensors for high performance in detection of molecular biomarkers: More rapid, sensitive, and universal. Biosens. Bioelectron..

[4] Hu Q., Yan J., Ren K. (2024). DNA Self-Assembly: A Tool to Improve Biochemical Reaction Performance. ACS Mater. Lett..

[5] Kiesling T., Cox K., Davidson E.A., Dretchen K., Grater G., Hibbard S., Lasken R.S., Leshin J., Skowronski E., Danielsen M. (2007). Sequence specific detection of DNA using nicking endonuclease signal amplification (NESA). Nucleic Acids Res..

[6] Jang E.K., Yang M., Pack S.P. (2015). Highly-efficient T4 DNA ligase-based SNP analysis using a ligation fragment containing a modified nucleobase at the end. Chem. Commun..

[7] Völker J., Gindikin V., Breslauer K.J. (2024). Higher-Order DNA Secondary Structures and Their Transformations: The Hidden Complexities of Tetrad and Quadruplex DNA Structures, Complexes, and Modulatory Interactions Induced by Strand Invasion Events. Biomolecules.

[8] Zhou S., Yuan L., Hua X., Xu L., Liu S. (2015). Signal amplification strategies for DNA and protein detection based on polymeric nanocomposites and polymerization: A review. Anal. Chim. Acta.

[9] Wen D., Liu Q., Cui Y., Kong J., Yang H., Liu Q. (2018). DNA based click polymerization for ultrasensitive IFN-γ fluorescent detection. Sens. Actuator B-Chem..

[10] Chen K., Shen Z., Wang G., Gu W., Zhao S., Lin Z., Liu W., Cai Y., Mushtaq G., Jia J. (2022). Research progress of CRISPR-based biosensors and bioassays for molecular diagnosis. Front. Bioeng. Biotechnol..

[11] Zhou Y., Tang L., Lyu J., Shiyi L., Liu Q., Pang R., Li W., Guo X., Zhong X., He H. (2024). A dual signal amplification system with specific signal identification for rapid and sensitive detection of miRNA. Talanta.

[12] Huang G., Li C., Wu R., Xue G., Song Q., Lan L., Xue C., Xu L., Shen Z. (2024). Self-assembly of protein-DNA hybrids dedicated to an accelerated and self-primed strand displacement amplification for reinforced serum microRNA probing. Anal. Chim. Acta.

[13] Patel M., Agrawal M., Srivastava A. (2022). Signal amplification strategies in electrochemical biosensors via antibody immobilization and nanomaterial-based transducers. Mater. Adv..

[14] Zhou X., Schuh D.A., Castle L.M., Furst A.L. (2022). Recent advances in signal amplification to improve electrochemical biosensing for infectious diseases. Front. Chem..

[15] Chen A., Zhuo Y., Chai Y., Yuan R. (2019). Bipedal DNA walker mediated enzyme-free exponential isothermal signal amplification for rapid detection of microRNA. Chem. Commun..

[16] Chen Y., Wang Q., Xu J., Xiang Y., Yuan R., Chai Y. (2013). A new hybrid signal amplification strategy for ultrasensitive electrochemical detection of DNA based on enzyme-assisted target recycling and DNA supersandwich assemblies. Chem. Commun..

[17] Zambry N.S., Awang M.S., Beh K.K., Hamzah H.H., Bustami Y., Obande G.A., Khalid M.F., Ozsoz M., Abd Manaf A., Aziah I. (2023). A label-free electrochemical DNA biosensor used a printed circuit board gold electrode (PCBGE) to detect SARS-CoV-2 without amplification. Lab Chip.

[18] Li X., Song T., Chen Z., Shi X., Chen C., Zhang Z. (2015). A universal fast colorimetric method for DNA signal detection with DNA strand displacement and gold nanoparticles. J. Nanomater..

[19] Pedrero M., Campuzano S., Pingarrón J.M. (2011). Electrochemical genosensors based on PCR strategies for microorganisms detection and quantification. Anal. Methods.

[20] Giakoumaki E., Minunni M., Tombelli S., Tothill I.E., Mascini M., Bogani P., Buiatti M. (2003). Combination of amplification and post-amplification strategies to improve optical DNA sensing. Biosens. Bioelectron..

[21] Gu L., Yan W., Liu L., Wang S., Zhang X., Lyu M. (2018). Research progress on rolling circle amplification (RCA)-based biomedical sensing. Pharmaceuticals.

[22] Yao C., Zhang R., Tang J., Yang D. (2021). Rolling circle amplification (RCA)-based DNA hydrogel. Nat. Protoc..

[23] Soroka M., Wasowicz B., Rymaszewska A. (2021). Loop-mediated isothermal amplification (LAMP): The better sibling of PCR?. Cells.

[24] He H., Zhou Y., Chen B., Zhang Y., Zhong X., Xu L., Guo B., Yin C., Zhou X., Li Q. (2023). Nucleic acid amplification with specific signal filtration and magnification for ultrasensitive colorimetric detection. Talanta.

[25] Tang Z., Sun F., Chai Y., Zhang C., Wang J., Li H. (2023). Intelligent dual-drive DNA nanosensor for ultrasensitive detection of prostate cancer-related circulating microRNA-200c. Microchem J..

[26] Li Y., Lucci T., Villarruel Dujovne M., Jung J.K., Capdevila D.A., Lucks J.B. (2025). A cell-free biosensor signal amplification circuit with polymerase strand recycling. Nat. Chem. Biol..

[27] Park K.S., Choi A., Park T.-I., Pack S.P. (2024). Fluorometric and Colorimetric Method for SARS-CoV-2 Detection Using Designed Aptamer Display Particles. Biosensors.

[28] Jayasena S.D. (1999). Aptamers: An emerging class of molecules that rival antibodies in diagnostics. Clin. Chem..

[29] Park T.-I., Yang A.H., Kanth B.K., Pack S.P. (2025). Aptamers as Diagnostic and Therapeutic Agents for Aging and Age-Related Diseases. Biosensors.

[30] Wu L., Zhang Y., Wang Z., Zhang Y., Zou J., Qiu L. (2022). Aptamer-Based cancer cell analysis and treatment. ChemistryOpen.

[31] Bujold K.E., Lacroix A., Sleiman H.F. (2018). DNA nanostructures at the interface with biology. Chem.

[32] Hui L., Bai R., Liu H. (2022). DNA-based nanofabrication for nanoelectronics. Adv. Funct. Mater..

[33] Bae W., Kocabey S., Liedl T. (2019). DNA nanostructures in vitro, in vivo and on membranes. Nano Today.

[34] Wang D.-X., Wang J., Wang Y.-X., Du Y.-C., Huang Y., Tang A.-N., Cui Y.-X., Kong D.-M. (2021). DNA nanostructure-based nucleic acid probes: Construction and biological applications. Chem. Sci..

[35] Nicolson F., Ali A., Kircher M.F., Pal S. (2020). DNA nanostructures and DNA-functionalized nanoparticles for cancer theranostics. Adv. Sci..

[36] Kaminski M.M., Abudayyeh O.O., Gootenberg J.S., Zhang F., Collins J.J. (2021). CRISPR-based diagnostics. Nat. Biomed. Eng.

[37] Wang Y., Qian J., Shi T., Wang Y., Ding Q., Ye C. (2024). Application of extremophile cell factories in industrial biotechnology. Enzyme Microb. Technol..

[38] Jang E.K., Lee M.J., Kim J., Lee J.W., Pack S.P. (2023). Noise-reduced nicking enzyme-based isothermal amplification via blocking the 3′-end of the amplicon using a novel fluorophore-immobilized binder. Sens. Actuator B-Chem..

[39] Kumar M., Nandeshwar R., Lad S.B., Megha K., Mangat M., Butterworth A., Knapp C.W., Knapp M., Hoskisson P.A., Corrigan D.K. (2021). Electrochemical sensing of SARS-CoV-2 amplicons with PCB electrodes. Sens. Actuator B-Chem..

[40] Jiang K., Wu J., Qiu Y., Go Y.Y., Ban K., Park H.J., Lee J.-H. (2021). Plasmonic colorimetric PCR for Rapid molecular diagnostic assays. Sens. Actuator B-Chem..

[41] Lee H.G., Choi W., Yang S.Y., Kim D.-H., Park S.-G., Lee M.-Y., Jung H.S. (2021). PCR-coupled paper-based surface-enhanced Raman scattering (SERS) sensor for rapid and sensitive detection of respiratory bacterial DNA. Sens. Actuator B-Chem..

[42] Li Z., Zou S., Wu S., Miao X., Ma D.-L. (2021). Polymerase chain reaction-based ultrasensitive detection of HBV DNA via G-quadruplex selective iridium (III) complex luminescent probe. Talanta.

[43] Li M., Li D., Huang G., Zhou L., Wen Q., Zhu W., Pan H. (2021). Signal-on electrochemical DNA (E-DNA) sensor for accurate quantification of nicking-assisted rolling circle amplification (N-RCA) products with attomolar sensitivity. Anal. Methods.

[44] Luo F., Xiang G., Pu X., Yu J., Chen M., Chen G. (2015). A novel ultrasensitive ECL sensor for DNA detection based on nicking endonuclease-assisted target recycling amplification, rolling circle amplification and hemin/g-quadruplex. Sensors.

[45] Chang D., Li J., Liu R., Liu M., Tram K., Schmitt N., Li Y. (2023). A Colorimetric Biosensing Platform with Aptamers, Rolling Circle Amplification and Urease-Mediated Litmus Test. Angew. Chem.-Int. Edit..

[46] Chen F., Lu W., Din L., Li F.-R. (2024). A novel RCA-based DNA sensor system for specific and quantitative detection of Klebsiella pneumonia. Microchem J..

[47] Zhang Y., Wang W., Zhou X., Lin H., Zhu X., Lou Y., Zheng L. (2024). CRISPR-Responsive RCA-Based DNA Hydrogel Biosensing Platform with Customizable Signal Output for Rapid and Sensitive Nucleic Acid Detection. Anal. Chem..

[48] Zou J., Hu J., Shen Y., Zhang L., Bai W., Wang L., Li J., Yan L., Zhang Z., Bai H. (2025). ISFET Biosensor with Loop-Mediated Isothermal Amplification for Electronic Rapid Detection of Mycoplasma Pneumoniae. Sensors.

[49] Li Y., Xue H., Fei Y., Yang Y., Huang D., Wang L., Xiong X., Xiong X. (2023). A rapid and closed-tube method based on the dual-color fluorescence loop-mediated isothermal amplification for visual detection of Atlantic salmon (*Salmo salar*). Food Chem..

[50] Sen A., Masetty M., Weerakoon S., Morris C., Yadav J.S., Apewokin S., Trannguyen J., Broom M., Priye A. (2024). based loop-mediated isothermal amplification and CRISPR integrated platform for on-site nucleic acid testing of pathogens. Biosens. Bioelectron..

[51] Zhu H., Zhang H., Xu Y., Laššáková S., Korabečná M., Neužil P. (2020). PCR past, present and future. Biotechniques.

[52] Kuang H., Ma W., Xu L., Wang L., Xu C. (2013). Nanoscale superstructures assembled by polymerase chain reaction (PCR): Programmable construction, structural diversity, and emerging applications. Accounts Chem. Res..

[53] Waters D.L., Shapter F.M. (2014). The polymerase chain reaction (PCR): General methods. Cereal Genom. Methods Protoc..

[54] Brongersma M.L., Halas N.J., Nordlander P. (2015). Plasmon-induced hot carrier science and technology. Nat. Nanotechnol..

[55] Ali M.M., Li F., Zhang Z., Zhang K., Kang D.-K., Ankrum J.A., Le X.C., Zhao W. (2014). Rolling circle amplification: A versatile tool for chemical biology, materials science and medicine. Chem. Soc. Rev..

[56] Yue S., Li Y., Qiao Z., Song W., Bi S. (2021). Rolling circle replication for biosensing, bioimaging, and biomedicine. Trends Biotechnol..

[57] Levine C., Hiasa H., Marians K.J. (1998). DNA gyrase and topoisomerase IV: Biochemical activities, physiological roles during chromosome replication, and drug sensitivities. Biochim. Biophys. Acta, Gene Struct. Expr..

[58] Drummond T.G., Hill M.G., Barton J.K. (2003). Electrochemical DNA sensors. Nat. Biotechnol..

[59] Fapohunda F.O., Qiao S., Pan Y., Wang H., Liu Y., Chen Q., Lü P. (2022). CRISPR Cas system: A strategic approach in detection of nucleic acids. Microbiol. Res..

[60] Li Y., Li S., Wang J., Liu G. (2019). CRISPR/Cas systems towards next-generation biosensing. Trends Biotechnol..

[61] Tomita N., Mori Y., Kanda H., Notomi T. (2008). Loop-mediated isothermal amplification (LAMP) of gene sequences and simple visual detection of products. Nat. Protoc..

[62] Yang N., Zhang H., Han X., Liu Z., Lu Y. (2024). Advancements and applications of loop-mediated isothermal amplification technology: A comprehensive overview. Front. Microbiol..

[63] Garg N., Ahmad F.J., Kar S. (2022). Recent advances in loop-mediated isothermal amplification (LAMP) for rapid and efficient detection of pathogens. Curr. Res. Microb. Sci..

[64] Bai H., Liu Y., Gao L., Wang T., Zhang X., Hu J., Ding L., Zhang Y., Wang Q., Wang L. (2024). A portable all-in-one microfluidic device with real-time colorimetric LAMP for HPV16 and HPV18 DNA point-of-care testing. Biosens. Bioelectron..

[65] Tian W., Li P., He W., Liu C., Li Z. (2019). Rolling circle extension-actuated loop-mediated isothermal amplification (RCA-LAMP) for ultrasensitive detection of microRNAs. Biosens. Bioelectron..

[66] Hu Z., Chen J., Li Y., Wang Y., Zhang Q., Hussain E., Yang M., Shahzad S.A., Yu D., Yu C. (2017). Nucleic acid-controlled quantum dots aggregation: A label-free fluorescence turn-on strategy for alkaline phosphatase detection. Talanta.

[67] Ji X., Lin X., Rivnay J. (2023). Organic electrochemical transistors as on-site signal amplifiers for electrochemical aptamer-based sensing. Nat. Commun..

[68] Lim J., Son S.U., Ki J., Kim S., Lee J., Jang S., Seo S.B., Jang H., Kang T., Jung J. (2024). Dual structure-switching aptamer-mediated signal amplification cascade for SARS-CoV-2 detection. Biosens. Bioelectron..

[69] Idili A., Parolo C., Alvarez-Diduk R., Merkoçi A. (2021). Rapid and efficient detection of the SARS-CoV-2 spike protein using an electrochemical aptamer-based sensor. ACS Sens..

[70] Yu Z.-g., Lai R.Y. (2018). A reagentless and reusable electrochemical aptamer-based sensor for rapid detection of ampicillin in complex samples. Talanta.

[71] Idili A., Gerson J., Parolo C., Kippin T., Plaxco K.W. (2019). An electrochemical aptamer-based sensor for the rapid and convenient measurement of l-tryptophan. Anal. Bioanal. Chem..

[72] Asai K., Yamamoto T., Nagashima S., Ogata G., Hibino H., Einaga Y. (2020). An electrochemical aptamer-based sensor prepared by utilizing the strong interaction between a DNA aptamer and diamond. Analyst.

[73] Downs A.M., Gerson J., Leung K.K., Honeywell K.M., Kippin T., Plaxco K.W. (2022). Improved calibration of electrochemical aptamer-based sensors. Sci. Rep..

[74] Guo W., Zhang C., Ma T., Liu X., Chen Z., Li S., Deng Y. (2021). Advances in aptamer screening and aptasensors’ detection of heavy metal ions. J. Nanobiotechnol..

[75] Abu-Ali H., Nabok A., Smith T.J. (2019). Development of novel and highly specific ssDNA-aptamer-based electrochemical biosensor for rapid detection of mercury (II) and lead (II) ions in water. Chemosensors.

[76] Kim H., Surwade S.P., Powell A., O’Donnell C., Liu H. (2014). Stability of DNA origami nanostructure under diverse chemical environments. Chem. Mat..

[77] Williamson P., Piskunen P., Ijäs H., Butterworth A., Linko V., Corrigan D.K. (2023). Signal amplification in electrochemical DNA biosensors using target-capturing DNA origami tiles. ACS Sens..

[78] Wang T., Zheng X., Chai H., Miao P. (2024). DNA nanostructure disintegration-assisted SPAAC ligation for electrochemical biosensing. Nano Lett..

[79] Wen Y., Pei H., Shen Y., Xi J., Lin M., Lu N., Shen X., Li J., Fan C. (2012). DNA nanostructure-based interfacial engineering for PCR-free ultrasensitive electrochemical analysis of microRNA. Sci. Rep..

[80] Pei H., Lu N., Wen Y., Song S., Liu Y., Yan H., Fan C. (2010). A DNA nanostructure-based biomolecular probe carrier platform for electrochemical biosensing. Adv. Mater..

[81] Li H., Han M., Weng X., Zhang Y., Li J. (2021). DNA-tetrahedral-nanostructure-based entropy-driven amplifier for high-performance photoelectrochemical biosensing. ACS Nano.

[82] Shan L., Chen Y., Tan X., Ge S., Zhang L., Li L., Yu J., Li L. (2023). Tetrahedral DNA nanostructure-engineered paper-based sensor with an enhanced antifouling ability for photoelectrochemical sensing. Anal. Chem..

[83] Zhang K., Huang W., Huang Y., Li H., Wang K., Zhu X., Xie M. (2019). DNA tetrahedron based biosensor for Argonaute2 assay in single cells and human immunodeficiency virus type-1 related ribonuclease H detection in vitro. Anal. Chem..

[84] Liu X., He F., Zhang F., Zhang Z., Huang Z., Liu J. (2020). Dopamine and melamine binding to gold nanoparticles dominates their aptamer-based label-free colorimetric sensing. Anal. Chem..

[85] Gupta R., Kumar A., Kumar S., Pinnaka A.K., Singhal N.K. (2021). Naked eye colorimetric detection of Escherichia coli using aptamer conjugated graphene oxide enclosed Gold nanoparticles. Sens. Actuator B-Chem..

[86] Zong C., Liu J. (2019). The arsenic-binding aptamer cannot bind arsenic: Critical evaluation of aptamer selection and binding. Anal. Chem..

[87] Li C., Hu X., Lu J., Mao X., Xiang Y., Shu Y., Li G. (2018). Design of DNA nanostructure-based interfacial probes for the electrochemical detection of nucleic acids directly in whole blood. Chem. Sci..

[88] Zhao L., Suo Z., Liu Y., Wei M., Jin H. (2023). An amplifiable ratiometric fluorescent aptasensor for aflatoxin B1 detection based on dendrimer-like DNA nanostructures coupled with catalytic hairpin self-assembly. Sens. Actuator B-Chem..

[89] Wang M., Lv Z., Liu Y., Wei M. (2025). Simultaneous detection of ochratoxin A and aflatoxin B1 based on stable tuning fork-shaped DNA fluorescent aptasensor. J. Fluoresc..

[90] Wang S., Zhang L., Wan S., Cansiz S., Cui C., Liu Y., Cai R., Hong C., Teng I.-T., Shi M. (2017). Aptasensor with expanded nucleotide using DNA nanotetrahedra for electrochemical detection of cancerous exosomes. ACS Nano.

[91] Wang Y., Duan H., Yalikun Y., Cheng S., Li M. (2024). Chronoamperometric interrogation of an electrochemical aptamer-based sensor with tetrahedral DNA nanostructure pendulums for continuous biomarker measurements. Anal. Chim. Acta.

[92] Wang T., Zhang Z., Chai H., Liu X., Miao P. (2025). Electrochemical Aptasensing of Vascular Endothelial Growth Factor by Construction of DNA Triangular Pyramid Frustum Reaction Interface. ACS Electrochem..

[93] Walter H.-K., Bauer J., Steinmeyer J., Kuzuya A., Niemeyer C.M., Wagenknecht H.-A. (2017). “DNA origami traffic lights” with a split aptamer sensor for a bicolor fluorescence readout. Nano Lett..

[94] Nameghi M.A., Danesh N.M., Ramezani M., Hassani F.V., Abnous K., Taghdisi S.M. (2016). A fluorescent aptasensor based on a DNA pyramid nanostructure for ultrasensitive detection of ochratoxin A. Anal. Bioanal. Chem..

[95] Jing C., Chen H., Cai R., Tian Y., Zhou N. (2020). An electrochemical aptasensor for ATP based on a configuration-switchable tetrahedral DNA nanostructure. Anal. Methods.

[96] Ding J., Gu Y., Li F., Zhang H., Qin W. (2015). DNA nanostructure-based magnetic beads for potentiometric aptasensing. Anal. Chem..

[97] Chauhan N., Xiong Y., Ren S., Dwivedy A., Magazine N., Zhou L., Jin X., Zhang T., Cunningham B.T., Yao S. (2022). Net-shaped DNA nanostructures designed for rapid/sensitive detection and potential inhibition of the SARS-CoV-2 virus. J. Am. Chem. Soc..

[98] Chen Y., Chen X., Zhang B., Zhang Y., Li S., Liu Z., Gao Y., Zhao Y., Yan L., Li Y. (2024). DNA framework signal amplification platform-based high-throughput systemic immune monitoring. Signal Transduct. Target. Ther..

[99] Lacroix A., Sleiman H.F. (2021). DNA nanostructures: Current challenges and opportunities for cellular delivery. ACS Nano.

[100] Seeman N.C., Sleiman H.F. (2017). DNA nanotechnology. Nat. Rev. Mater..

[101] Mathur D., Medintz I.L. (2019). The growing development of DNA nanostructures for potential healthcare-related applications. Adv. Healthc. Mater..

[102] Abu-Salah K.M., Zourob M.M., Mouffouk F., Alrokayan S.A., Alaamery M.A., Ansari A.A. (2015). DNA-based nanobiosensors as an emerging platform for detection of disease. Sensors.

[103] Abu-Salah K.M., Alrokyan S.A., Khan M.N., Ansari A.A. (2010). Nanomaterials as analytical tools for genosensors. Sensors.

[104] Wen Z.-B., Peng X., Yang Z.-Z., Zhuo Y., Chai Y.-Q., Liang W.-B., Yuan R. (2019). A dynamic 3D DNA nanostructure based on silicon-supported lipid bilayers: A highly efficient DNA nanomachine for rapid and sensitive sensing. Chem. Commun..

[105] Pei H., Wan Y., Li J., Hu H., Su Y., Huang Q., Fan C. (2011). Regenerable electrochemical immunological sensing at DNA nanostructure-decorated gold surfaces. Chem. Commun..

[106] Jang E.K., Koike Y., Ide Y., Tajima K., Kanaori K., Pack S.P. (2020). Nucleobase-involved native chemical ligation: A novel reaction between an oxanine nucleobase and N-terminal cysteine for oligonucleotide–peptide conjugation. Chem. Commun..

[107] Jang E.K., Ki M.-R., Pack S.P. (2017). Design of reactive-end DNA oligomers via incorporation of oxanine into oligonucleotides using terminal deoxynucleotidyl transferase. Process Biochem..

[108] Jang E.K., Son R.G., Pack S.P. (2019). Novel enzymatic single-nucleotide modification of DNA oligomer: Prevention of incessant incorporation of nucleotidyl transferase by ribonucleotide-borate complex. Nucleic Acids Res..

[109] Domsicova M., Korcekova J., Poturnayova A., Breier A. (2024). New insights into aptamers: An alternative to antibodies in the detection of molecular biomarkers. Int. J. Mol. Sci..

[110] Song S., Wang L., Li J., Fan C., Zhao J. (2008). Aptamer-based biosensors. Trac-Trends Anal. Chem..

[111] Park K.S., Choi A., Kim H.J., Park I., Eom M.-S., Yeo S.-G., Son R.G., Park T.-I., Lee G., Soh H.T. (2023). Ultra-sensitive label-free SERS biosensor with high-throughput screened DNA aptamer for universal detection of SARS-CoV-2 variants from clinical samples. Biosens. Bioelectron..

[112] Park K.S., Cha H., Niu J., Soh H.T., Lee J.H., Pack S.P. (2024). DNA-controlled protein fluorescence: Design of aptamer-split peptide hetero-modulator for GFP to respond to intracellular ATP levels. Nucleic Acids Res..

[113] Wang Z., Yang X., Lee N.Z., Cao X. (2022). Multivalent aptamer approach: Designs, strategies, and applications. Micromachines.

[114] Park K.S., Park T.-I., Lee J.E., Hwang S.-Y., Choi A., Pack S.P. (2024). Aptamers and Nanobodies as new bioprobes for SARS-CoV-2 diagnostic and therapeutic system applications. Biosensors.

[115] Wang G., Dong H., Han J., Zhang M., Huang J., Sun J., Guan F., Shen Z., Xu D., Sun X. (2022). Interference-resistant aptasensor with tetrahedral DNA nanostructure for profenofos detection based on the composites of graphene oxide and polyaniline. Bioelectrochemistry.

[116] Yang F., Li J., Dong H., Wang G., Han J., Xu R., Kong Q., Huang J., Xiang Y., Yang Q. (2022). A novel label-free electrochemiluminescence aptasensor using a tetrahedral DNA nanostructure as a scaffold for ultrasensitive detection of organophosphorus pesticides in a luminol–H_2_O_2_ system. Analyst.

[117] Huang Z., Wang D., Zhang Q., Zhang Y., Peng R., Tan W. (2024). Leveraging aptamer-based DNA nanotechnology for bioanalysis and cancer therapeutics. Accounts Mater. Res..

[118] Ma W., Zhan Y., Zhang Y., Mao C., Xie X., Lin Y. (2021). The biological applications of DNA nanomaterials: Current challenges and future directions. Signal Transduct. Target. Ther..

[119] Suo Z., Liang X., Jin H., He B., Wei M. (2021). A signal-enhancement fluorescent aptasensor based on the stable dual cross DNA nanostructure for simultaneous detection of OTA and AFB 1. Anal. Bioanal. Chem..

[120] Liu M., Dong J., Suo Z., Wang Q., Wei M., He B., Jin H. (2023). A convenient fluorescent/electrochemical dual-mode biosensor for accurate detection of Pb2+ based on DNAzyme cycle. Bioelectrochemistry.

[121] Artés J.M., Li Y., Qi J., Anantram M., Hihath J. (2015). Conformational gating of DNA conductance. Nat. Commun..

[122] Fahlman R.P., Sen D. (2002). DNA conformational switches as sensitive electronic sensors of analytes. J. Am. Chem. Soc..

[123] Mao D., Li W., Liu X., Chen J., Wei D., Luo L., Yuan Q., Yang Y., Zhu X., Tan W. (2025). Rolling-circle-amplification-based DNA-enzyme nanostructure for immobilization and functionalization of enzymes. Chem.

[124] Bai H., Bu S., Liu W., Wang C., Li Z., Hao Z., Wan J., Han Y. (2020). An electrochemical aptasensor based on cocoon-like DNA nanostructure signal amplification for the detection of Escherichia coli O157: H7. Analyst.

[125] Tian R., Sun J., Ye Y., Lu X., Wang W., Sun X. (2024). Ultrasensitive Aptasensor for α-Amatoxin Detection Based on the DNA Tetrahedral Nanostructure Triggering Rolling Circle Amplification and Signal Amplification. J. Agric. Food Chem..

